# Effects of suppressing bioavailability of insulin‐like growth factor on age‐associated intervertebral disc degeneration

**DOI:** 10.1002/jsp2.1112

**Published:** 2020-07-27

**Authors:** Rebecca Kritschil, Zhongying Zhang, Changbin Lei, Jiongbiao Zhong, Qing Dong, Joon Lee, Cheryl A. Conover, Gwendolyn Sowa, Abbe N. Vallejo, Nam Vo

**Affiliations:** ^1^ Department of Orthopedic Surgery University of Pittsburgh Pittsburgh Pennsylvania USA; ^2^ Department of Orthopedic Surgery Kobe University Graduate School of Medicine Kobe Japan; ^3^ Department of Orthopaedic Surgery First Affiliated Hospital of Jinan University Guangdong China; ^4^ Department of Spinal Surgery The First Affiliated Hospital of University of South China Hengyang Hunan P.R.China; ^5^ Endocrine Research Unit Mayo Clinic Rochester Minnesota USA; ^6^ Department of Physical Medicine and Rehabilitation University of Pittsburgh Pittsburgh Pennsylvania USA; ^7^ Department of Pediatrics, UPMC Children's Hospital of Pittsburgh University of Pittsburgh Pittsburgh Pennsylvania USA

**Keywords:** aging, cellular senescence, IGF‐1, intervertebral disc degeneration, PAPPA, proteoglycan

## Abstract

Suppression of the insulin‐like growth factor‐1 (IGF‐1) signaling pathway reduces age‐related disorders and increases lifespan across species, making the IGF‐1 pathway a key regulator of aging. Previous in vitro intervertebral disc cell studies have reported the pro‐anabolic effect of exogenously adding IGF‐1 on matrix production. However, the overall effects of suppressing IGF‐1 signaling on age‐related intervertebral disc degeneration (IDD) is not known. Here, the effects of suppressing IGF‐1 signaling on age‐related IDD in vivo were examined using *PAPPA* ^−/−^ mice. These are animals with targeted deletion of pregnancy‐associated plasma protein A (PAPPA), the major protease that cleaves inhibitory IGF binding proteins that control bioavailability of IGF‐1 for cell signaling. Compared to age‐matched wild‐type (Wt) littermates, reduced levels of matrix proteoglycan (PG) and aggrecan were seen in discs of 23‐month old *PAPPA* ^*−/−*^ mice. Decreased aggrecanolysis and expression of two key catabolic markers, matrix metalloproteinase‐3 and a disintegrin and metalloproteinase with thrombospondin motifs‐4, were also observed in discs of old *PAPPA* ^*−/−*^ mice compared to Wt littermates. Suppressing IGF‐1 signaling has been implicated to shift cellular metabolism toward maintenance rather than growth and decreasing cellular senescence. Along this line, discs of old *PAPPA* ^*−/−*^ mice also exhibited lower cellular senescence, assessed by p53 and lamin B1 markers. Collectively, the data reveal complex regulation of disc matrix homeostasis by PAPPA/IGF‐1 signaling during chronologic aging, that is, reduced IGF‐1 bioavailability confers the benefit of decreasing disc cellular senescence and matrix catabolism but also the disadvantage of decreasing disc PG matrix anabolism. This pathway requires further mechanistic elucidation before IGF‐1 could be considered as a therapeutic growth factor for treating IDD.

## Graphical abstract

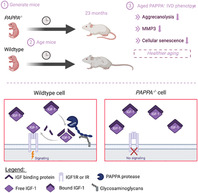

## 1 INTRODUCTION

Growth factors are peptides working in an autocrine or paracrine manner to regulate cellular activity by binding to cell surface receptors and initiating downstream signaling in target cells. Insulin‐like growth factors (IGF) are involved in proliferation and function of nearly every cell, tissue, and organ in the body. IGF‐1 is one of two signaling proteins in the IGF family that binds to IGF‐1 receptor (IGF‐1R). IGF‐1 signaling is a well‐established modulator of aging.^1^ Genetic and pharmacologic suppression of the IGF‐1 signaling pathway in numerous animal models has consistently shown that decreasing IGF‐1 signaling leads to healthier aging and increased lifespan.^1^, ^2^, ^3^, ^4^ Mutations known to impair IGF‐1 receptor function, such as natural polymorphisms of the *IGF1R* gene, and other genetic alterations that affect IGF‐1 signaling, have been found in numerous populations of centenarians around the world from Japan to New England.^1^, ^5^, ^6^ The exact mechanisms of how the IGF‐1 pathway increases longevity in mammals is unknown, highlighting the complexity of this signaling pathway. However, it has been suggested that decreasing IGF‐1 signaling reduces mTOR and shifts cellular metabolism from cell growth to cell maintenance and repair activities, decreasing the accumulation of senescent cells.^6^, ^7^, ^8^ Despite some inconsistent findings on the role of IGF‐1 Among human centenarian and animal model studies,^1^, ^9^ there is overwhelming evidence to support that disruptions to the IGF‐1 signaling pathway promotes healthy longevity.^1^, ^5^, ^6^, ^8^, ^10^

Intervertebral disc degeneration (IDD) can be initiated by an imbalance in disc matrix homeostasis, leading to a pathologic catabolic state where more extracellular matrix is being degraded than what can be produced by disc cells. IDD can lead to biomechanical instability and a range of spine problems, including low back pain.^11^, ^12^, ^13^, ^14^, ^15^ Growth factors such as IGF‐1 are an attractive biologic therapeutic for treating IDD because they are able to stimulate anabolic production of extracellular matrix.^11^, ^16^, ^17^, ^18^ Early studies on disc cell culture and IGF‐1 stimulation showed increased proteoglycan (PG) synthesis of nucleus pulposus (NP) cells in bovine and canine models.^16^, ^17^ More recently, IGF‐1 has been shown to stimulate both DNA synthesis and downstream signaling of bovine NP and annulus fibrosus (AF) cells in vitro.^19^ Additionally, another *in vitro* study showed the beneficial effect of IGF‐1 on decreasing senescent cells in human AF cells exposed to hydrogen peroxide, suggesting a protective effect of IGF‐1 against oxidative stress that is typically observed in aging disc.^20^ The growing research interest in the role of IGF‐1 in IDD has given rise to the idea that IGF‐1 can be used as a therapeutic to treat IDD.

The therapeutic potential of IGF‐1 for treating IDD, however, is complex and unclear. There is evidence supporting the use of IGF‐1 to treat IDD patients in vivo based on reported decreased levels of serum IGF‐1 in IDD patients. These studies have correlated low circulating IGF‐1 levels with increased level of IDD.^21^, ^22^ However, circulating IGF‐1 level is also influenced by age, sex, and BMI, all of which influence the risk of developing IDD.^23^, ^24^, ^25^, ^26^ Therefore, adjustments for these factors in control vs patient groups are imperative for correctly evaluating IGF‐1 levels in control subjects vs patients with IDD. Likewise, circulating IGF‐1 levels do not reflect individual tissue concentrations. Free unbound IGF‐1 or IGF‐1 bioavailability, in the disc specifically, is a better measure of the activity of the GH/IGF‐1 pathway in relation to IDD rather than total serum or total protein expression level, which are the methods used almost exclusively in previous papers studying IGF‐1 and IDD.

Aging is a well‐established major risk factor of IDD as discs appear to undergo age‐related degenerative changes earlier in life than other tissues.^12^ However, the role of IGF‐1 signaling in regulating age‐related IDD is unknown. On the one hand, in vitro disc studies suggest that increasing IGF‐1 signaling promotes disc health through stimulating matrix production. On the other hand, decreasing IGF‐1 signaling in vivo has been shown to mediate age‐related disorders and increase lifespan. In light of these conflicting findings, this study investigated how IGF‐1 signaling regulates disc aging in vivo using *PAPPA* ^−/−^ mice.^27^ These are animals with targeted deletion of pregnancy‐associated plasma protein A (*PAPPA*), a gene encoding a protease that specifically cleaves three of the IGF binding proteins—IGFBP‐2, ‐4, and ‐5—that sequester IGF‐1 and reduce its bioavailability.^28^ The PAPPA amino acid sequence contains five short consensus repeats in its C terminus. Two of these repeats, SCR3 and SCR4, are glycosylated and mediate the binding of PAPPA to cell surface.^28^ Because of its location at the cell surface, PAPPA is responsible for the local IGF‐1 bioavailability and IGF‐1 signaling activity more than the circulating level of IGF‐1. The mean lifespan for *PAPPA* ^−/−^ mice is approximately 40% greater than wild‐type (Wt) mice.^1^, ^4^, ^28^, ^29^ In this study, the effects of reduced IGF‐1 bioavailability on disc matrix homeostasis and age‐related IDD in 23‐month old *PAPPA* ^*−/−*^ mice and their Wt littermates were investigated.

## 2 METHODS

### 2.1 Mice

Creation, genotype, and genetic screening of *PAPPA* ^−/−^ mice and their Wt littermates have been reported elsewhere.^27^ Gene profiles and pathological assessments over the lifespan have also been reported.^4^, ^30^ Animals were reared in specific‐pathogen free facility. For this study, 23‐month‐old mice were used, their spines collected and analyzed. Six‐month‐old Wt mice were also included in analysis of disc aggrecanolysis. The number of animals used in each experiment are as indicated in the figure legends. All studies were performed according to animal research protocols approved by Institutional Animal Care and Use Committees of the University of Pittsburgh.

### 2.2 Aggrecanolysis

To measure disc aggrecan fragmentation, seven caudal discs were pooled to prepare extract for immunoblots using a previously established method.^31^ Anti‐aggrecan primary antibody (1:1000 dilution, Abcam ab36861, Cambridge, United Kingdom) and anti‐rabbit horse radish peroxidase secondary antibody (1:5000 dilution, Thermo Scientific PI‐31460, Waltham, MA, USA), were used for Western blot detection of aggrecan proteolytic fragments.

### 2.3 Western blotting

Protein from caudal discs of seven mice were extracted using Tissue Protein Extraction Reagent (T‐PER) with proteinase inhibitor cocktail per the manufacturer's instructions (Thermo Fisher 78510, Waltham, MA). Western blotting for the expression of p53, matrix metalloproteinase‐3 (MMP‐3), and a disintegrin and metalloproteinase with thrombospondin motifs‐4 (ADAMTS‐4) was also performed. Primary antibodies (1:1000 dilution) to p53 (Cell Signaling Technology 2524, Danvers, MA), MMP‐3 (Abcam ab52915, Cambridge, United Kingdom), ADAMTS‐4 (Abcam ab185722, Cambridge, United Kingdom), and β‐actin (Thermo Fisher PA1‐183, Waltham, MA) and secondary anti‐rabbit horse radish peroxidase antibody (1:5000 dilution, Thermo Fisher 31460, Waltham, MA) were used.

Following electrophoresis on Tris‐HEPES 4%‐20% gradient polyacrylamide denaturing gel (Thermo Scientific 25204, Waltham, MA), separated proteins were transferred to a polyvinylidene difluoride membrane. Signals were measured using chemiluminescent detection (Thermo Scientific 34096, Waltham, MA) and ChemiDoc MP system (Bio‐Rad, Hercules, CA). The protein bands were analyzed using NIH Image J 1.44p with local background subtraction. The data obtained were normalized with β‐actin.

### 2.4 Histology

Isolated lumbar disc tissues from three *PAPPA* ^−/−^ and Wt mice were fixed and decalcified in Decacifier I solution (Leica 3800440) at 4°C overnight. All tissues were dehydrated through a graded alcohol series and then embedded in paraffin (Tissue Tek processor and Leica Embedder) and cut into three 4‐μm thick sections in the coronal plane. The sections were stained with safranin‐O and fast green dyes by standard procedures and photographed under ×40 to ×200 magnification (Nikon Eclipse E 800).

### 2.5 Immunohistochemistry

Detection of aggrecan and lamin B1 were performed on deparaffinized coronal disc tissue sections of 4‐μm thickness. The sections were pretreated with chondroitinase ABC degrades chondroitin sulfate A, chondroitin sulfate B, and chondroitin sulfate C (0.25 U/mL; Sigma C3667, St. Louis, MO) at 37°C for 1‐hour to unmask epitopes. Endogenous peroxidase activity was eliminated by the treatment with 3% hydrogen peroxide, and then incubated 15 minutes with Avidin D solution and 15 minutes with biotin solution (Vector Laboratories SP‐2001, Burlingame, CA). Permeabilization and blocking was done with 10% goat serum, 1% bovine serum albumin, and 0.25% Triton‐X 100 in phosphate buffered saline for 30 minutes. Primary antibody against Aggrecan (1:200; EMD Millipore AB1031) and lamin B1(1:1000; Abcam ab16048, Cambridge, United Kingdom) were applied at 4°C overnight, then thoroughly washed with washing buffer (PBS + 0.05% Tween20). Biotinylated goat anti‐rabbit secondary antibody (1:200; Vector Laboratories BA‐1000, Burlingame, CA) was applied for 30 minutes and the sections were washed again in washing buffer, followed by the avidin‐biotin amplification (Vector Laboratories PK‐6100, Burlingame, CA) for 30 minutes. AEC substrate/chromogen KIT (ScyTek Laboratories ACG500) was used for 10 minutes. Hematoxylin was used as a counterstain. All mounted slides with mounting medium Cytoseal 60 (Thermo Fisher Scientific 8310‐4, Waltham, MA) were visualized on a Nikon Eclipse E800 microscope.

### 2.6 DMMB assay for total glycosaminoglycan

PG content of disc NP from Wt and *PAPPA* ^−/−^ mice was measured using 1,9‐dimethylmethylene blue buffer (DMMB) assay for total glycosaminoglycan (GAG). NP tissue from three lumbar discs of each mouse was isolated using the dissecting microscope and stored at −80°C. The tissue was digested in papain (Sigma–Aldrich P4762, St. Louis, MO) under standard methods.^32^ Samples were prepared in DMMB (Sigma–Aldrich 341088, St. Louis, MO), and the optical density was read at 540 nm and compared with established standards for chondroitin sulfate (Sigma–Aldrich C‐8529, St. Louis, MO). The GAG content was normalized based upon DNA concentration of each sample; this was measured using the Pico green dsDNA quantitation kit (Thermo Fisher Scientific P7589, Waltham, MA).

### 2.7 Statistical analysis

Data are expressed as the mean ± SD of three independent samples. For Western blot and total GAG analysis, 95% confidence intervals were calculated to determine statistical significance at *P* < .05 level. The confidence intervals were calculated based on the *t*‐distribution because of the small sample size.^33^

## 3 RESULTS

### 3.1 Aged *PAPPA* ^*−/−*^ mice exhibit decreased disc PG matrix content

**FIGURE 1:**
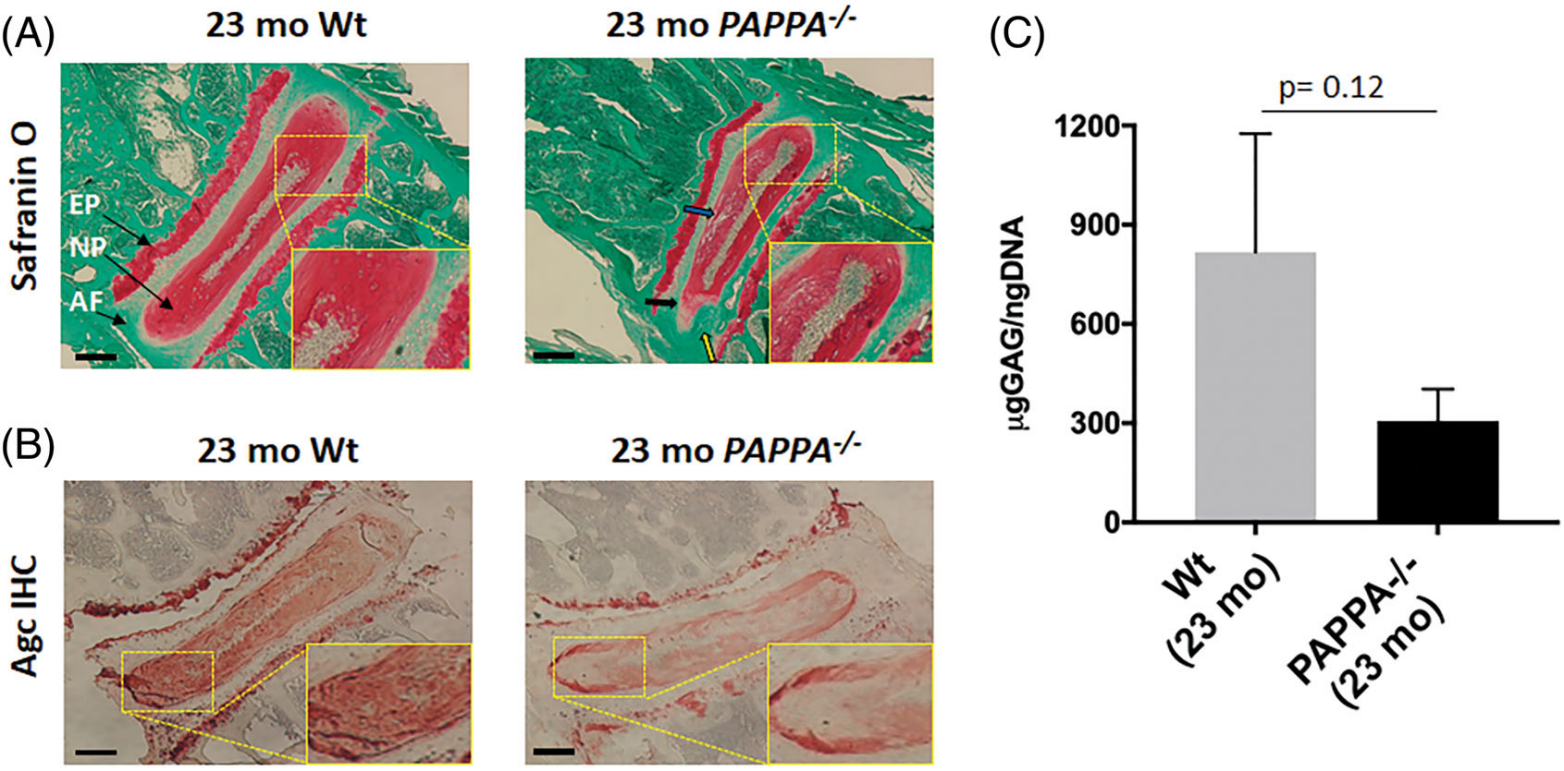
Assessment of disc proteoglycan matrix in 23‐month‐old *PAPPA* ^*−/−*^ compared to 23‐month‐old Wt mice. Histology and immunohistochemistry slides were prepared as described in methods. Representative images are shown. A, Histology showing less safranin‐O/fast green staining of PG in disc of 23‐month‐old *PAPPA* ^*−/−*^ mice than Wt littermates. Compared to Wt control, discs of *PAPPA* ^*−/−*^ mice exhibited slightly greater loss of NP cellularity, loss of well‐defined AF‐NP boundary (black arrow), loss of NP matrix as indicated by large empty space gaps (blue arrow), and loss of AF structure in which the AF lamellae become less concentric and more serpentine with each lamella spaced farther apart (yellow arrow). B, Aggrecan immunohistochemistry showing less IHC staining for aggrecan in the nucleus pulposus and endplate of 23‐month‐old *PAPPA* ^*−/−*^ mice, the than aged Wt mice. ×40 magnification, scale bar = 200 μm for images shown in panels (A) and (B). C, DMMB quantitative assay for sulfated GAG normalized to the amount of DNA. The level of GAGs in aged *PAPPA* ^*−/−*^ mice was about 2.5‐fold less than aged Wt littermates, but the difference is not statistically significant (*P* = .12). Abbreviations: AF, annulus fibrosus; disc, intervertebral disc (n = 3); EP, endplate; GAG, glycosaminoglycans; NP, nucleus pulposus; PG, proteoglycan; Wt, wild‐type

Progressive loss of disc PG matrix occurs with age. To determine whether decreasing IGF‐1 signaling in the disc influences the amount of disc PG in old mice, safranin‐O/fast green histological staining was performed. The intensity of safranin‐O staining was reduced in the lumbar discs of 23‐month‐old *PAPPA* ^*−/−*^ mice compared to their Wt littermates (Figure 1A). Disc histological scoring also reveal slightly higher AF fissure formation and greater loss of NP matrix and NP‐AF boundary in *PAPPA* ^*−/−*^ mice compared to their Wt control (Figure 1A), although these differences are not statistically different (Table 1). GAGs are covalently attached to PGs and are responsible for providing the osmotic properties necessary for normal disc tissue compression. This functional ability is dependent on a high concentration of GAGs and a decrease in GAGs is a hallmark of both disc aging and IDD. Consistent with the safranin‐O histological staining, DMMB assay for quantitative measurement of GAG content, a surrogate marker for PG, revealed 2.6‐fold less disc GAG in aged *PAPPA* ^*−/−*^ mice compared to aged Wt littermates (Figure 1C). Immunohistochemical (IHC) staining of aggrecan, a major PG in the disc, was reduced in the NP and endplate regions in discs of aged *PAPPA* ^*−/−*^ mice compared to their Wt littermates (Figure 1B). Taken together, these qualitative and quantitative measurements show decreased PG content in aged mice discs with decreased IGF‐1 signaling (*PAPPA* ^*−/−*^) compared to Wt. These findings are consistent with the previous studies that reported the pro‐anabolic effects of IGF‐1 on matrix production in disc cell culture.^16^, ^34^, ^35^

**TABLE 1:** Disc histological scores

Score (mean ± SD)	Old Wt (23 mo)	Old *PAPPA* ^−/−^ (23 mo)
NP cellularity loss	1.1 ± 0.8	2.0 ± 1.0
NP clefts/fissures	1.1 ± 0.8	1.2 ± 0.7
AF structure	1.3 ± 0.7	1.4 ± 0.7
AF clefts/fissures	1.0 ± 0.6	1.2 ± 0.8
AF/NP boundary	1.3 ± 0.8	1.5 ± 1.1
Composite score	5.8 ± 1.7	7.3 ± 1.9

Abbreviations: AF, annulus fibrosus; NP, nucleus pulposus; PAPPA, pregnancy‐associated plasma protein A; WT, wild‐type.

### 3.2 Aged *PAPPA* ^*−/−*^ mice had less fragmented aggrecan in disc

**FIGURE 2:**
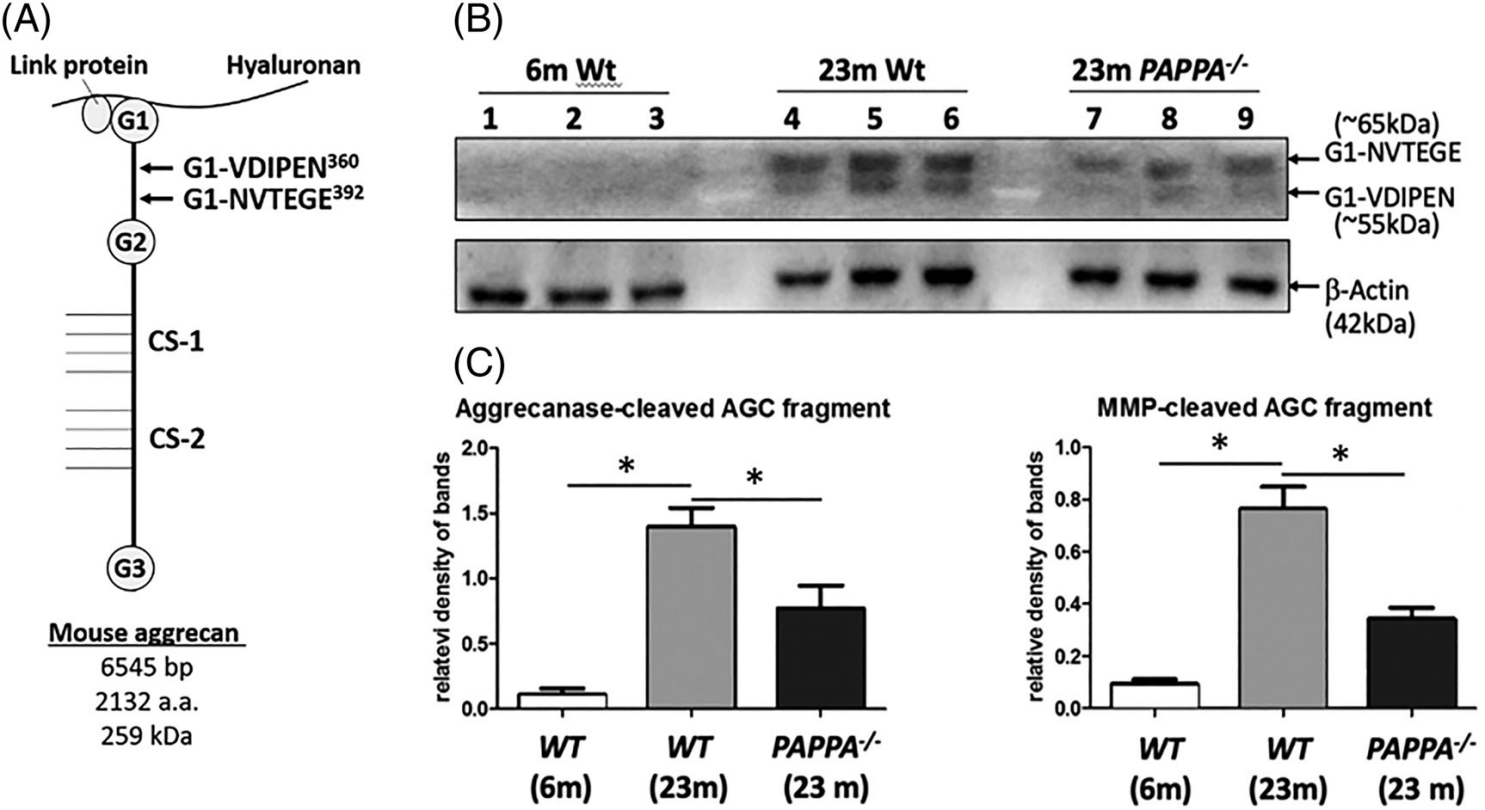
Assessment of catabolic activity in disc tissue of 23‐month‐old *PAPPA* ^*−/−*^ mice compared to 23‐month‐old Wt mice. Western blotting and densitometry measurements were performed as described in Section 2. A, A schematic of the mouse aggrecan core protein covalently linked to the sulphate‐rich GAG and noncovalently bound to a hyaluronan chain via the link protein. The cleavage sites between G1 and G2 interglobular domains by ADAMTS (G1‐NVTEGE392) and MMP (G1VDIPEN360) proteases are indicated. B, Western blot of 23‐month‐old *PAPPA* ^*−/−*^ and 23‐month‐old Wt disc tissues probed for aggrecan fragments. Six‐month‐old Wt tissues serve as young controls. Arrows indicate two specific cleavage products corresponding to ADAMTS (~65 kD) and MMP (~55 kD). Three replicates per condition. C, Quantification of aggrecan fragment western blot data for 6‐month‐old Wt, 23‐month‐old Wt, and 23‐month‐old *PAPPA* ^*−/−*^ tissues. Bars represent mean values of three different mouse tissues; error bars indicate SD. All values were normalized to β‐actin control. *, *P* < .05. Abbreviations: disc, intervertebral disc; MMP, matrix metalloproteinase; ADAMTS, a disintegrin and metalloproteinase with thrombospondin motifs; Wt, wild‐type

Since disc matrix homeostasis is determined by anabolic and catabolic matrix metabolism, decreased disc PG content in *PAPPA* ^*−/−*^ mice could be due to enhanced PG catabolism.^12^ With aging, disc cells gradually lose their capacity to synthesize new PG to replace what is degraded and lost over time, resulting in a net loss of disc PG.^12^ A schematic of mouse PG is pictured in Figure 2A, indicating the cleavage sites of MMPs and ADAMTS' between the G1 and G2 globular domains of the aggrecan core protein. G1‐VDIPEN360 and G1‐NVTEGE392 are G1‐bearing N‐terminal products generated by MMPs and ADAMTSs, respectively, which can be measured by Western blot. Six‐month‐old Wt mice were included as a young healthy control, which have little to no disc aggrecan fragmentation (Figure 2B,C). With age, there was a significant increase in the amount of disc aggrecan fragmentation from both MMPs and ADAMTS‐mediated cleavage within the interglobular domain of aggrecan (Figure 2B,C). Twenty‐three‐month‐old Wt mice had significantly more ADAMTS‐generated and MMP‐generated aggrecan fragments compared to 6‐month‐old Wt controls. However, in the aged 23‐month old *PAPPA* ^*−/−*^ mice, there were significant decreases in the amounts of aggrecan fragmentation from both MMPs and ADAMTS' compared to their Wt littermates (Figure 2B,C). Specifically, the amount of ADAMTS cleaved and MMP cleaved aggrecan in aged *PAPPA* ^*−/−*^ was about 2‐fold less than that seen in the Wt littermates. Therefore, decreasing IGF‐1 signaling reduces age‐related disc aggrecanolysis. This is consistent with findings that decreasing IGF‐1 signaling leads to healthier aging and less age‐related disorders.

### 3.3 Aged *PAPPA* ^*−/−*^ mice had lowered expression of disc catabolic markers, MMP‐3, and ADAMTS‐4

**FIGURE 3:**
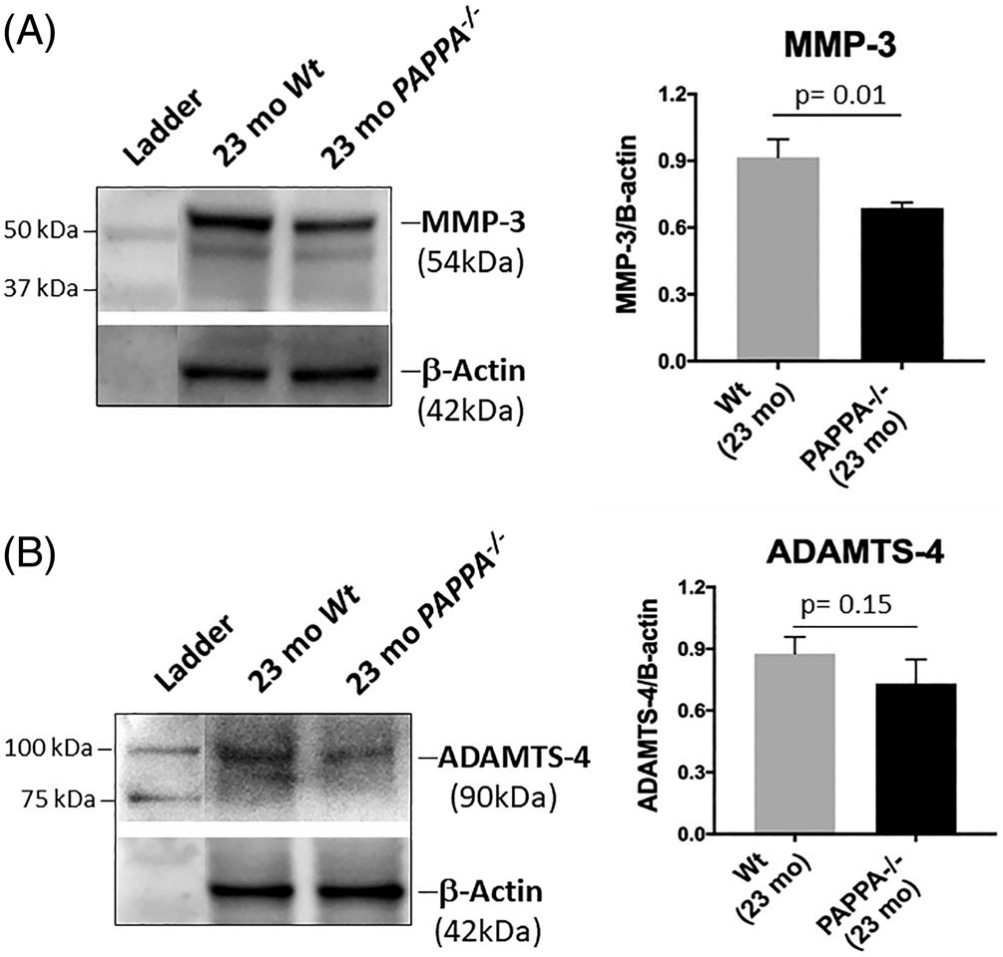
Assessment of catabolic protease expression in disc tissue of 23‐month‐old *PAPPA* ^*−/−*^ mice compared to 23‐month‐old Wt mice. Data shown are representative immunoblots and densitometric quantification of MMP‐3 (A) and ADAMTS‐4 (B) protein expression. Bars represent mean values of three different mouse tissues; error bars indicate SD, n = 3. All values were normalized to β‐actin control. *, *P* < .05

MMP‐3 and ADAMTS‐4 represent the major metalloproteinases found in disc tissue^12^ implicated in aggrecanolysis. To determine if these two proteases were regulated by IGF‐1 signaling, their protein expression levels in disc tissue were measured in *PAPPA* ^*−/−*^ mice by Western blot. There was a significant decrease in the level of MMP‐3 protein in aged *PAPPA* ^*−/−*^ mice compared to Wt mice littermates (Figure 3A). ADAMTS‐4 protein expression was also decreased in aged *PAPPA* ^*−/−*^ mice compared to Wt littermates, but this decrease was not significant. Compared to the significant decrease in aggrecan fragmentation seen in aged *PAPPA* ^*−/−*^ mice (Figure 2A,B), the modest decrease in protein expression of MMP‐3 and ADAMTS‐4 (Figure 3A,B) in *PAPPA* ^*−/−*^ mice relative to the Wt mice implies that IGF‐1 signaling may not directly influence protein expression of these two proteases in the disc. The mechanism for decreased disc aggrecanolysis seen in aged *PAPPA* ^*−/−*^ mice warrants further investigation.

### 3.4 Aged *PAPPA* ^*−/−*^ mice had increased disc lamin B1 and decreased p53 expression, indicating reduced disc cellular senescence

Senescent disc cells have recently been reported to exhibit a catabolic phenotype, called senescence‐associated secretory phenotype, and have imbalanced matrix homeostasis.^36^, ^37^ There is a strong correlation between the level of disc cellular senescence and grade of disc degeneration,^36^ and growing evidence suggests senescent disc cells are a key driver of age‐related IDD.^36^, ^37^ Therefore, the role of IGF‐1 in disc cellular senescence was investigated in aged *PAPPA* ^*−/−*^ mice. When a healthy cell becomes senescent, it develops certain characteristic morphological changes, which include enlarged and often irregular nuclei. Lamin B1 is a scaffolding component of the nuclear envelope, and its expression has been shown to be inversely correlated with cellular senescence.

**FIGURE 4:**
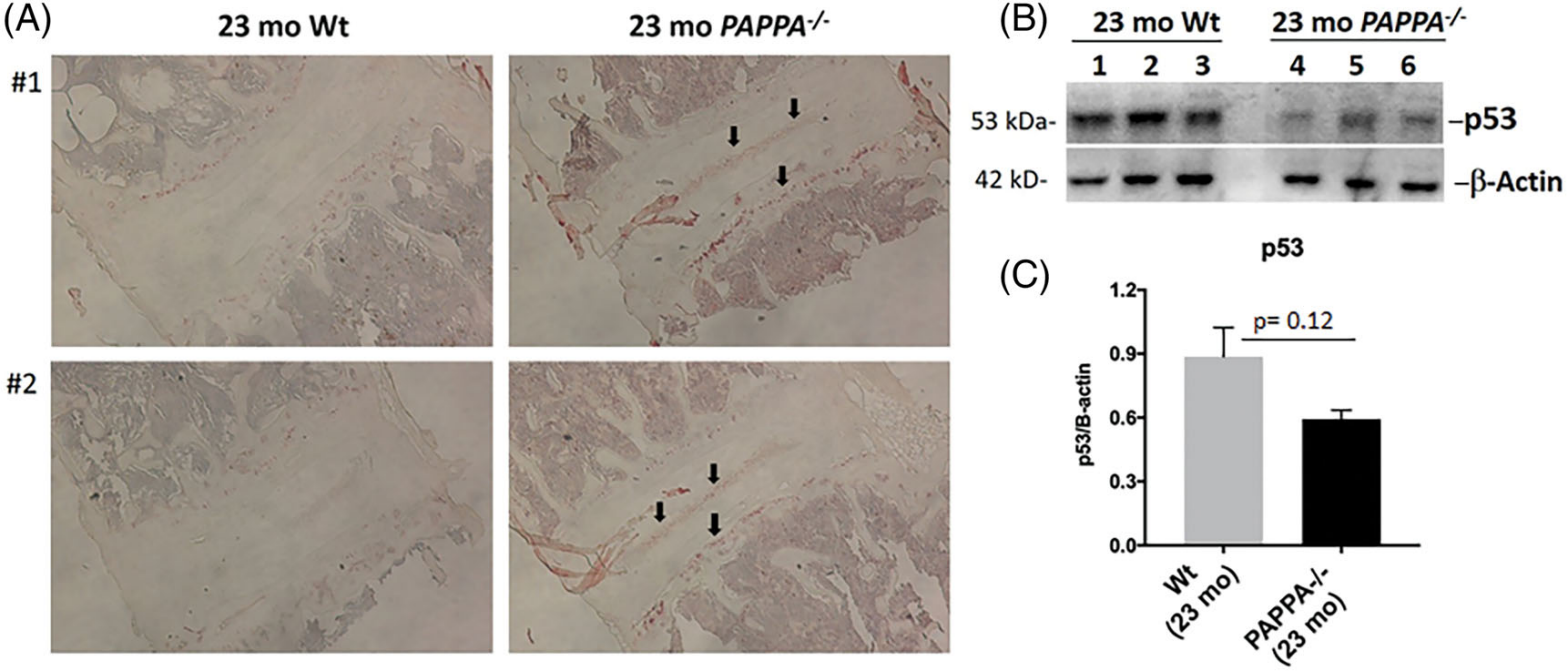
Assessment of disc cellular senescence in disc tissue of 23‐month‐old *PAPPA* ^*−/−*^ mice compared to 23‐month‐old Wt mice. Immunohistochemistry slides were prepared as described in Section 2. Representative examples are shown. A, Lamin B1 staining for qualitative assessment of senescent disc cells. There was higher intensity of lamin B1 staining in *PAPPA* ^*−/−*^ mice as indicated by the black arrows. No detectable lamin B1 staining was seen in 23‐month‐old Wt mice samples. B, Western blotting probed for p53 protein expression. Three replicates per condition. C, Densitometric quantification of p53 immunoblots of *PAPPA* ^*−/−*^ mice showed a decrease in p53 expression, albeit not statistically significant (*P* = .12), compared to 23‐month‐old Wt mice. Bars represent mean values of three different mouse tissues; error bars indicate SD. All values were normalized to β‐actin control

Compared to Wt littermates, aged *PAPPA* ^*−/−*^ mice had more nuclear lamin B1 IHC staining, as shown by the black arrows, indicating less senescent disc cells in aged *PAPPA* ^*−/−*^ mice (Figure 4A, right). Aged Wt mice had negligible levels of lamin B1 staining, as indicated by black arrows, suggesting higher number of senescent disc cells (Figure 4A, left). To validate this finding, disc protein level of another senescence biomarker, p53, was assessed by Western blot in aged *PAPPA* ^*−/−*^ mice and their Wt littermates. A major driver of cellular senescence is the accumulation of DNA damage, which induces p53 expression.^36^ Hence, greater p53 expression in a cell indicates greater likelihood of it progressing toward senescence.^36^ Compared to aged Wt mice, aged *PAPPA* ^*−/−*^ mice had less p53 protein expression, but this difference was not statistically significant (Figure 4C). Taken together, these data suggest aged *PAPPA* ^*−/−*^ mice have lower levels of disc cellular senescence compared to old Wt littermates.

## 4 DISCUSSION

Aging is a key contributor to IDD as disc matrix homeostasis becomes imbalanced with age, resulting in a net loss of PG. *PAPPA* ^*−/−*^ mice have reduced IGF‐1 bioavailability.^27^ Notably, their serum IGF‐1 levels in serum are not significantly different from Wt littermates, but they have significantly higher expression levels of inhibitory IGFBP4, the major substrate of PAPPA protein. As such *PAPPA* ^*−/−*^ mice have decreased IGF‐1 bioavailability. IGF1 protein level, when normalized to beta actin as assessedby Western blot, is similar in disc tissue of PAPPA−/− mice compared to Wt mice (Supplemental Fig S1), consistent with the known function of PAPPA in that its depletion is expected to decrease IGF1 bioavailability but not IGF1 protein expression or stability. Consistent with reports about the impact of deletions of components of the IGF system in other animal models, *PAPPA* ^*−/−*^ mice live on average about 40% longer compared to Wt mice.^4^ Here, disc matrix characteristics in aged *PAPPA* ^*−/−*^ mice was shown to be distinct from that of their Wt littermates. Specifically, the present data show that discs of aged *PAPPA* ^*−/−*^ mice exhibit overall reduced aging in the disc as reflected in decreased disc by cellular senescence and aggrecanolysis. These data are consistent with previous findings that *PAPPA* ^*−/−*^ mice have an overall healthier longevity with significantly reduced pathology at mid‐life and end‐of‐life.^30^ Thus, reducing IGF‐1 bioavailability in PAPPA^−/−^ mice had a positive effect in regulating disc matrix homeostasis during aging.

*PAPPA* ^*−/−*^ mice exhibit reduced disc matrix catabolism (Figure 2A,B). In the center of the disc, the gelatinous NP loses water content with age due to proteolytic degradation of aggrecan, the major PG in the disc.^12^ With increasing age, there is an increase in expression of disc proteases that break down aggrecan molecules for tissue remodeling in response to time‐dependent accumulation of injuries and damage. However, this becomes pathological when the remaining cells in the NP can no longer synthesize enough extracellular matrix (including aggrecan) to replace what is being degraded.^12^ Compared to Wt littermates, discs of aged *PAPPA* ^*−/−*^ mice exhibit significantly less aggrecanolysis (Figure 2), significantly decreased expression of the catabolic enzyme MMP‐3 (Figure 3), as well as lowered cellular senescence (Figure 4). These findings suggest that age‐related changes in the disc occurs more slowly in *PAPPA* ^*−/−*^ mice, which is consistent with their increased lifespan (40%) compared to that of Wt mice. In other words, *PAPPA* ^*−/−*^ mice age more slowly than their Wt counterpart and at 23 months of age, the discs of *PAPPA* ^*−/−*^ mice exhibit less PG catabolism and cellular senescence. It is also plausible that IGF‐1 regulates disc aggrecanolysis through its influences on cellular senescence, that is, decreased IGF‐1 signaling led to decreased disc aggrecanolysis through suppressing the development of disc cellular senescence. In congruence with this, it has recently been shown that senescent disc cells are catabolic, producing an abundant amount of MMPs that enhance aggrecanolysis on disc tissue.^37^, ^38^ Additionally, studies using human primary fibroblast IMR90 cells and mouse embryonic fibroblasts (MEFs) have reported that prolonged IGF‐1 treatment induces premature cellular senescence in a p53‐dependent manner.^39^ These findings support the idea that suppressed IGF‐1 signaling in *PAPPA* ^*−/−*^ mice leads to reduced disc cellular senescence which results in an overall decrease in disc aggrecanolysis. On the contrary, Gruber and coworkers reported that supplementing IGF‐1 in cell culture rescues AF cells from oxidative stress‐induced premature senescence.^20^ These conflicting findings in IGF‐1 regulation of disc cellular senescence might be due to the differences between their in vitro condition and our in vivo model as well as the potential metabolic differences between AF and NP cells in responding to stress. Additionally, IGF‐1 might also influence disc aggrecanolysis through the AKT pathway which has been implicated in regulating MMP expression,^40^ although confirmatory studies are needed.

The PG content in the disc of 23‐month‐old *PAPPA* ^*−/−*^ mice is less than that seen in 23‐month‐old Wt mice, as demonstrated by safranin‐O/fast green histology, DMMB assay for total GAG, and aggrecan IHC (Figure 1). This finding is consistent with the pro‐anabolic function of IGF‐1 as a growth factor, that is, less IGF‐1 signaling in *PAPPA* ^*−/−*^ mice leads to less matrix synthesis during development and less overall disc PG matrix. Previous work demonstrated IGF‐1 as an anabolic agent for discs able to promote both PG synthesis and disc cell proliferation.^11^, ^16^, ^17^, ^19^, ^34^, ^35^, ^41^ Concurrently, IGF‐1 also stimulated matrix production in chondrocytes. Loeser and coworkers reported significant increases in PG synthesis by chondrocytes derived from knees of osteoarthritis patients that were cultured in alginate beads with exogenous IGF‐1 (100‐1000 ng/mL).^42^ Increased PG synthesis in bovine monolayer articular chondrocytes and explant models stimulated with IGF‐1 were also documented.^34^, ^35^, ^41^, ^43^ Given the pro‐anabolic function of IGF‐1 on cells, it is possible that the low disc PG in *PAPPA* ^*−/−*^ mice is due to lack of anabolic stimulation from IGF‐1 since birth. Another possibility is *PAPPA* ^*−*/−^ mice have a decreased ability to synthesize new disc PG matrix as it is lost with age due to increased PG catabolism. The metabolic effects of IGF‐1 on disc PG matrix homeostasis.

IGF‐1 has been suggested as a therapeutic growth factor to stimulate PG synthesis to counteract the loss of disc PG with age and degeneration. However, the role of IGF‐1 in regulating disc matrix homeostasis is more complex than merely up regulating matrix production as reported in vitro studies. Our study comparing Wt and *PAPPA* ^*−/−*^ mice suggest that IGF‐1 signaling is also required for disc PG production in vivo, but prolonged signaling of IGF‐1 increases the risk of cellular senescence and aggrecanolysis, as seen in aged Wt mice. Furthermore, in order to synthesize new matrix following stimulation with IGF‐1, cells in the degenerated or aged disc must have the nutrients required to generate energy to perform these functions. However, the disc nutrient supply is decreased in most aged and degenerated discs due to end plate calcification.^44^, ^45^ Thus, IGF‐1 stimulation of matrix production under this limited nutrient status would likely induce bioenergetic stress in aged or degenerated discs. Adding to the complications of using exogenously administered IGF‐1 as a therapeutic approach for IDD patients, Le Maitre et al. found *IGFR1* expression in the ingrowing blood vessels that characterize part of the etiology in IDD.^46^ Therefore, adding IGF‐1 may exacerbate angiogenesis of the ingrowing blood vessels in degenerated discs. Lastly, Zhu et al. found higher levels of IGF‐1 in NP cells from lumbar disc herniation patients compared to controls, and a positive relationship between severity of LDH and IGF‐1 levels,^47^ which implicate detrimental effects of high IGF‐1 level in IDD. Given the observed complexity of IGF‐1 in modulating cellular metabolism and disc matrix homeostasis, more studies are needed before this signaling pathway can be considered as a therapeutic target to treat IDD. It should be noted that IGF‐1 action on the discs in our global PAPPA knockout mice could be due to its direct effects, that is, IGF‐1 entering disc tissue and stimulating disc cells, or indirect effects through impacting other body tissues which then affecting disc tissue via paracrine or endocrine pathways; it is not possible to differentiate between these two mechanisms, adding to the complexity of IGF‐1 action on disc biology that would require further investigation to elucidate. Nevertheless, this study suggests that decreasing global IGF‐1 bioavailability is overall beneficial for slowing down age‐associated IDD, but more temporal and spatial studies aimed at upregulating and downregulating IGF‐1 signaling are needed to further elucidate the mechanisms by which it impacts disc matrix homeostasis and age‐associated IDD.

## CONFLICT OF INTEREST

The authors declare no potential conflict of interest.

## AUTHOR CONTRIBUTIONS

**Rebecca Kritschil, Joon Lee, Gwendolyn Sowa, Abbe N. Vallejo,** and **Nam Vo**: Research design and/or data interpretation, Drafting and revising manuscript, read and approved the final submitted manuscript. **Zhongying Zhang, Changbin Lei, Jiongbiao Zhong, Qing Dong,** and **Cheryl A. Conover**: Data acquisition, read and approved the final submitted manuscript.

## Supporting information


**FIGURE S1** Assessment of IGF‐1 protein expression in disc tissue of 23‐month‐old *PAPPA* ^*−/−*^ mice compared to 23‐month‐old Wt mice. Data shown are representative IGF‐1 immunoblots (A) and densitometric quantification (B) Bars represent mean values of three different mouse tissues; error bars indicate SD, n = 3. All values were normalized to β‐actin control.
