# Supplementary figures and images for: Effects of suppressing bioavailability of insulin‐like growth factor on age‐associated intervertebral disc degeneration
Source: JOR Spine. 2020 Jul 27;3(4):e1112. doi: 10.1002/jsp2.1112 (PMC7770198; doi:10.1002/jsp2.1112)

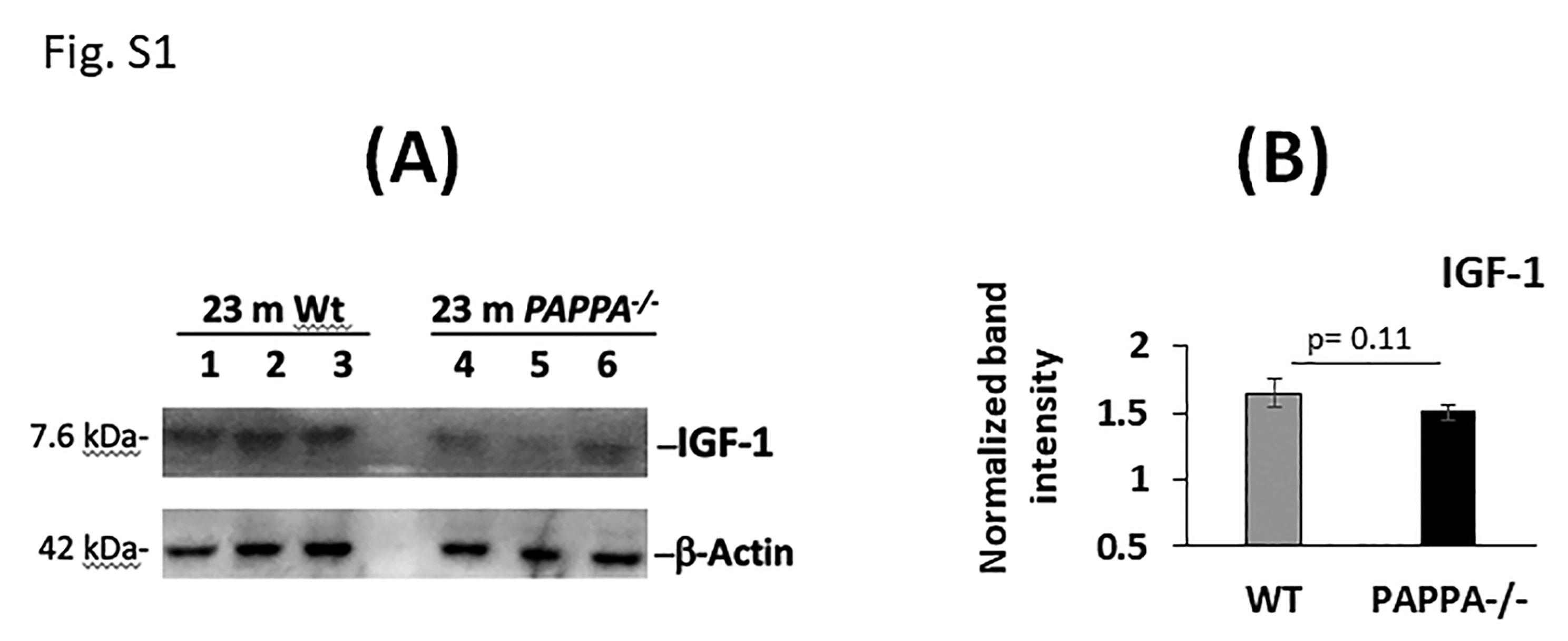

Supplement: Supplementary file 1 — FIGURE S1 Assessment of IGF‐1 protein expression in disc tissue of 23‐month‐old PAPPA −/− mice compared to 23‐month‐old Wt mice. Data shown are representative IGF‐1 immunoblots (A) and densitometric quantification (B) Bars represent mean values of three different mouse tissues; error bars indicate SD, n = 3. All values were normalized to β‐actin control. [file JSP2-3-e1112-s001.tif]
